# Supplementary material for: Synaptotagmin-11 facilitates assembly of a presynaptic signaling complex in post-Golgi cargo vesicles
Source: EMBO Rep. 2024 May 2;25(6):10. doi: 10.1038/s44319-024-00147-0 (PMC11169412; doi:10.1038/s44319-024-00147-0)
Supplement: Supplementary file 1 — Appendix [file 44319_2024_147_MOESM1_ESM.pdf]

# Appendix

## Table of content

**Appendix Figure S1** **Page 2**
**Normal kinetics of sEPSCs in cultured *Syt11*$^{-/-}$ hippocampal neurons**

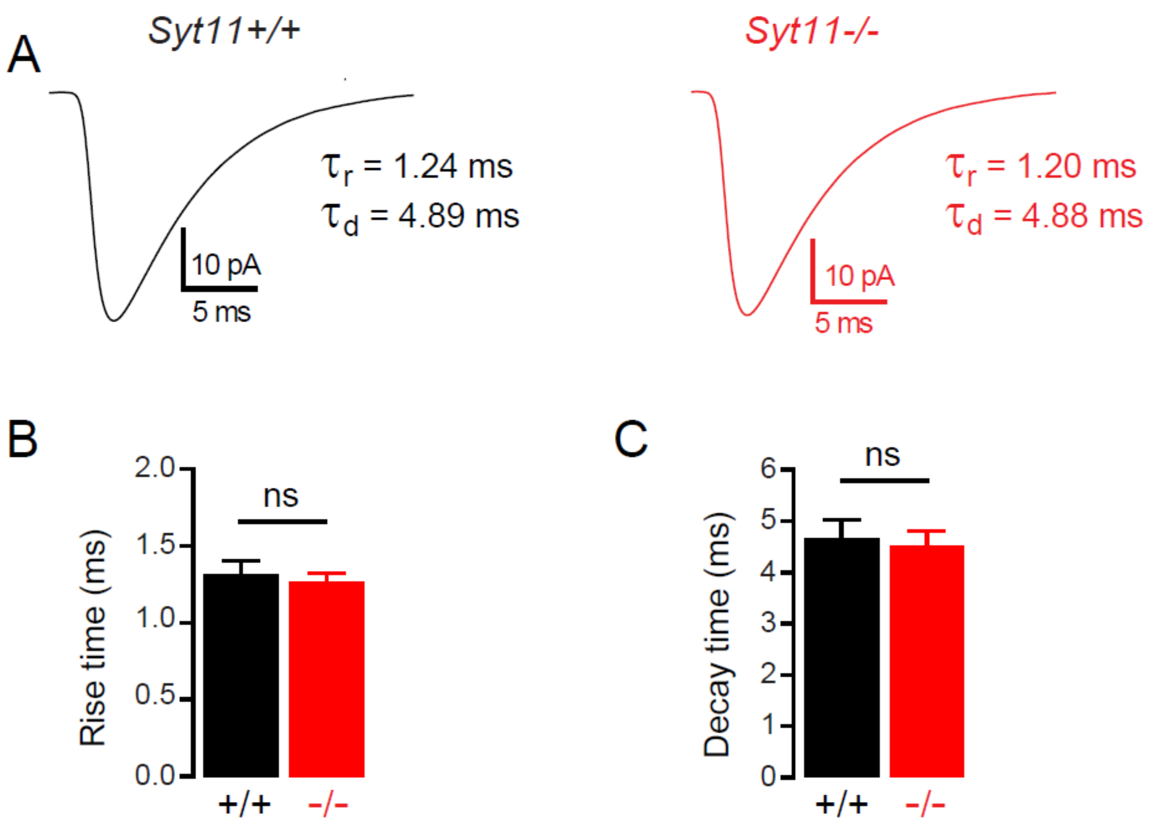


**Appendix Figure S1 - Normal kinetics of sEPSCs in cultured *Syt11*$^{-/-}$ hippocampal neurons**

(A) Representative average traces of sEPSCs from a *Syt11*$^{+/+}$ (black) and a *Syt11*$^{-/-}$ (red) neuron recorded at DIV15-19. Rise ($\tau_r$) and decay ($\tau_d$) times are indicated.

(B) Average sEPSC rise times per neuron (*Syt11*$^{+/+}$: 1.31 ± 0.11 ms vs *Syt11*$^{-/-}$: 1.26 ± 0.09 ms).

(C) Average sEPSC decay times per neuron (*Syt11*$^{+/+}$: 4.66 ± 0.36 ms vs *Syt11*$^{-/-}$: 4.52 ± 0.29 ms).

Data information: Data are presented as mean ± SEM. Statistical significance was determined by Mann-Whitney U test (B) or unpaired Student's *t*-test (C). ns not significant. *Syt11*$^{+/+}$, n =14 neurons; *Syt11*$^{-/-}$, n = 19 neurons from 6 preparations.
