# Supplementary material for: Synaptotagmin-11 facilitates assembly of a presynaptic signaling complex in post-Golgi cargo vesicles
Source: EMBO Rep. 2024 May 2;25(6):10. doi: 10.1038/s44319-024-00147-0 (PMC11169412; doi:10.1038/s44319-024-00147-0)
Supplement: Supplementary file 4 — Source data Fig. 1 [file 44319_2024_147_MOESM4_ESM.zip › EMBOR-2023-58002V2_SourceDataForFigure1/1B/1B bottom/README.pdf]

1B (bottom)

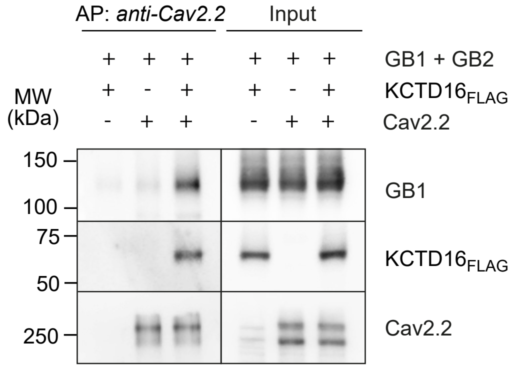
AP: anti-Cav2.2
Input
+ + + + + + GB1 + GB2
+ - + + - + KCTD16FLAG
- + + - + + Cav2.2
MW (kDa)
150
100
75
50
250
GB1
KCTD16FLAG
Cav2.2

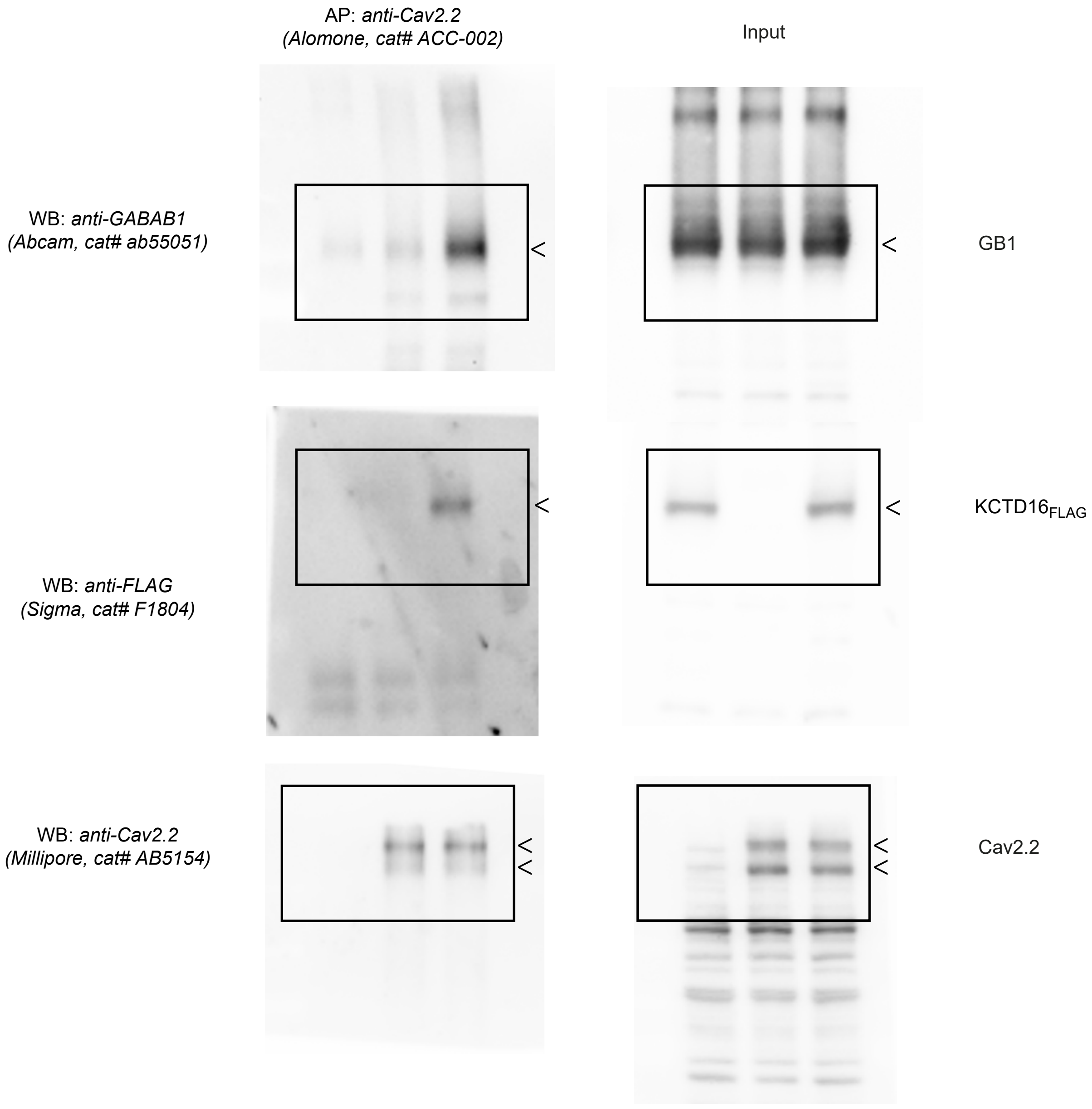
AP: anti-Cav2.2 (Alomone, cat# ACC-002)
Input
WB: anti-GABAB1 (Abcam, cat# ab55051)
GB1
WB: anti-FLAG (Sigma, cat# F1804)
KCTD16FLAG
WB: anti-Cav2.2 (Millipore, cat# AB5154)
Cav2.2
