# Supplementary material for: Synaptotagmin-11 facilitates assembly of a presynaptic signaling complex in post-Golgi cargo vesicles
Source: EMBO Rep. 2024 May 2;25(6):10. doi: 10.1038/s44319-024-00147-0 (PMC11169412; doi:10.1038/s44319-024-00147-0)
Supplement: Supplementary file 4 — Source data Fig. 1 [file 44319_2024_147_MOESM4_ESM.zip › EMBOR-2023-58002V2_SourceDataForFigure1/1B/1B top/README.pdf]

1B (top)

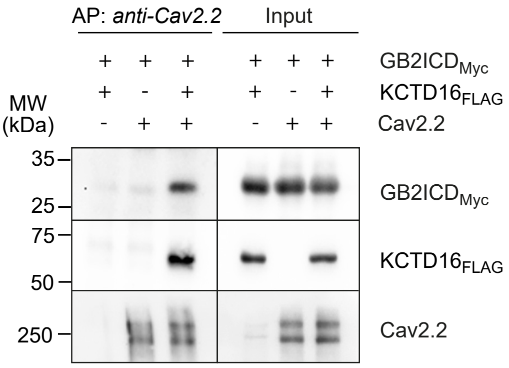

AP: anti-Cav2.2
Input
GB2ICDMyc
KCTD16FLAG
Cav2.2
MW (kDa)
35
25
75
50
250

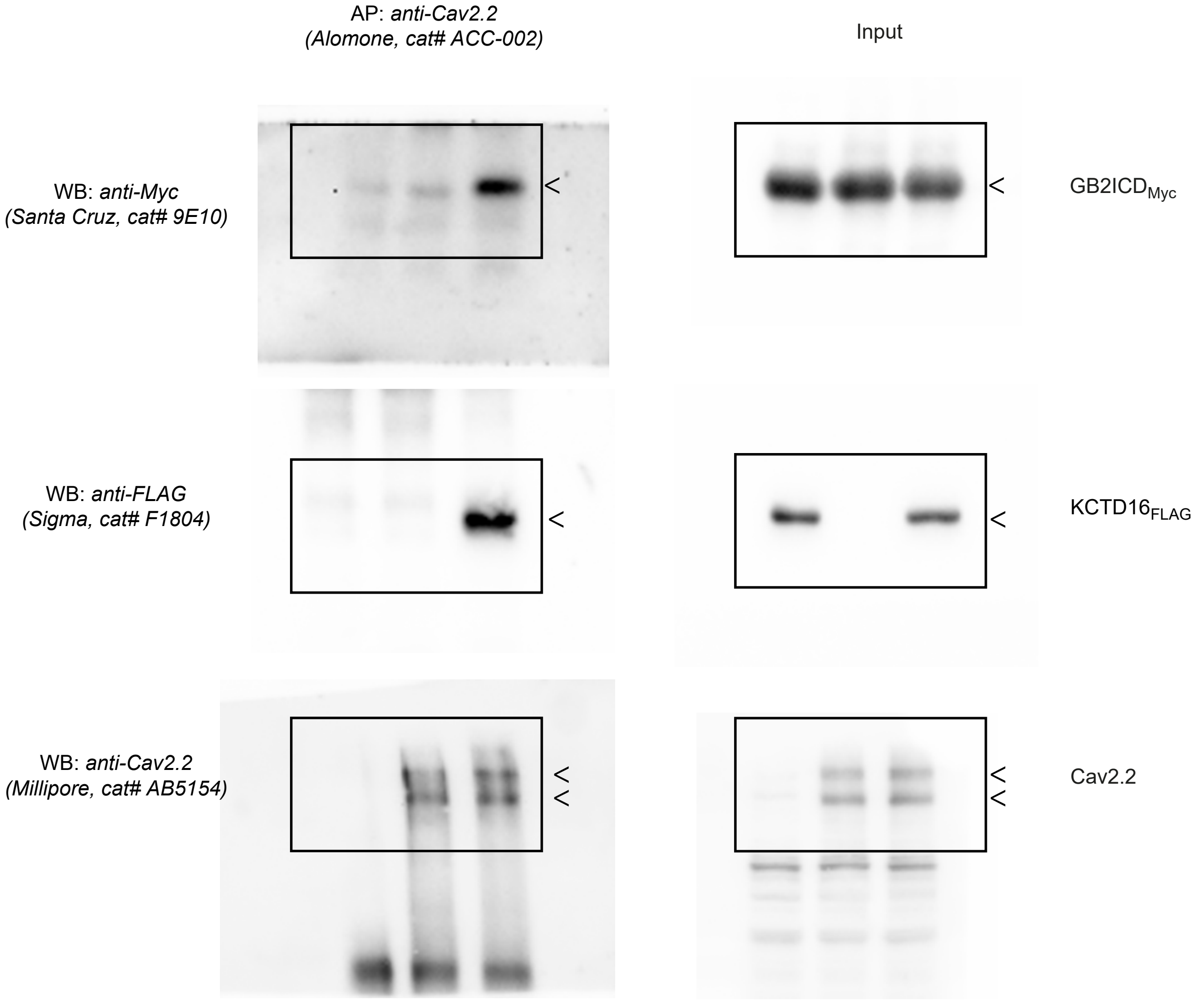

AP: anti-Cav2.2
(Alomone, cat# ACC-002)
Input
WB: anti-Myc
(Santa Cruz, cat# 9E10)
WB: anti-FLAG
(Sigma, cat# F1804)
WB: anti-Cav2.2
(Millipore, cat# AB5154)
GB2ICDMyc
KCTD16FLAG
Cav2.2
