# Supplementary material for: Synaptotagmin-11 facilitates assembly of a presynaptic signaling complex in post-Golgi cargo vesicles
Source: EMBO Rep. 2024 May 2;25(6):10. doi: 10.1038/s44319-024-00147-0 (PMC11169412; doi:10.1038/s44319-024-00147-0)
Supplement: Supplementary file 4 — Source data Fig. 1 [file 44319_2024_147_MOESM4_ESM.zip › EMBOR-2023-58002V2_SourceDataForFigure1/1C/README.pdf]

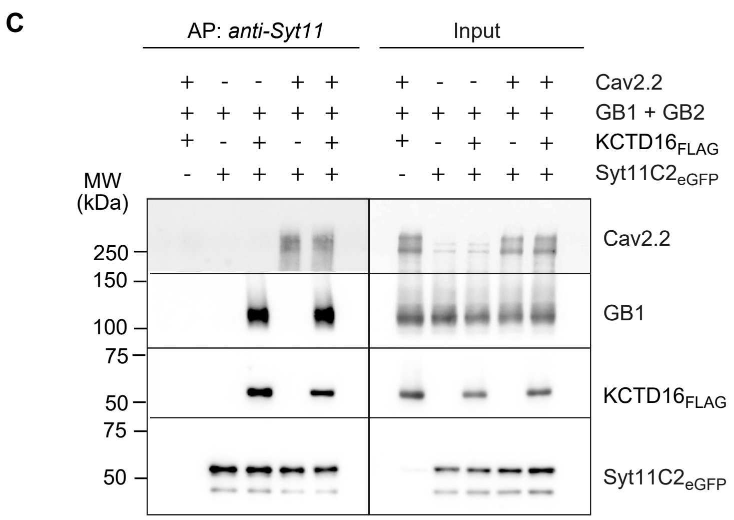
C
AP: anti-Syt11
Input
Cav2.2
GB1 + GB2
$KCTD16_{FLAG}$
$Syt11C2_{eGFP}$
MW (kDa)
250
150
100
75
50
75
50
Cav2.2
GB1
$KCTD16_{FLAG}$
$Syt11C2_{eGFP}$

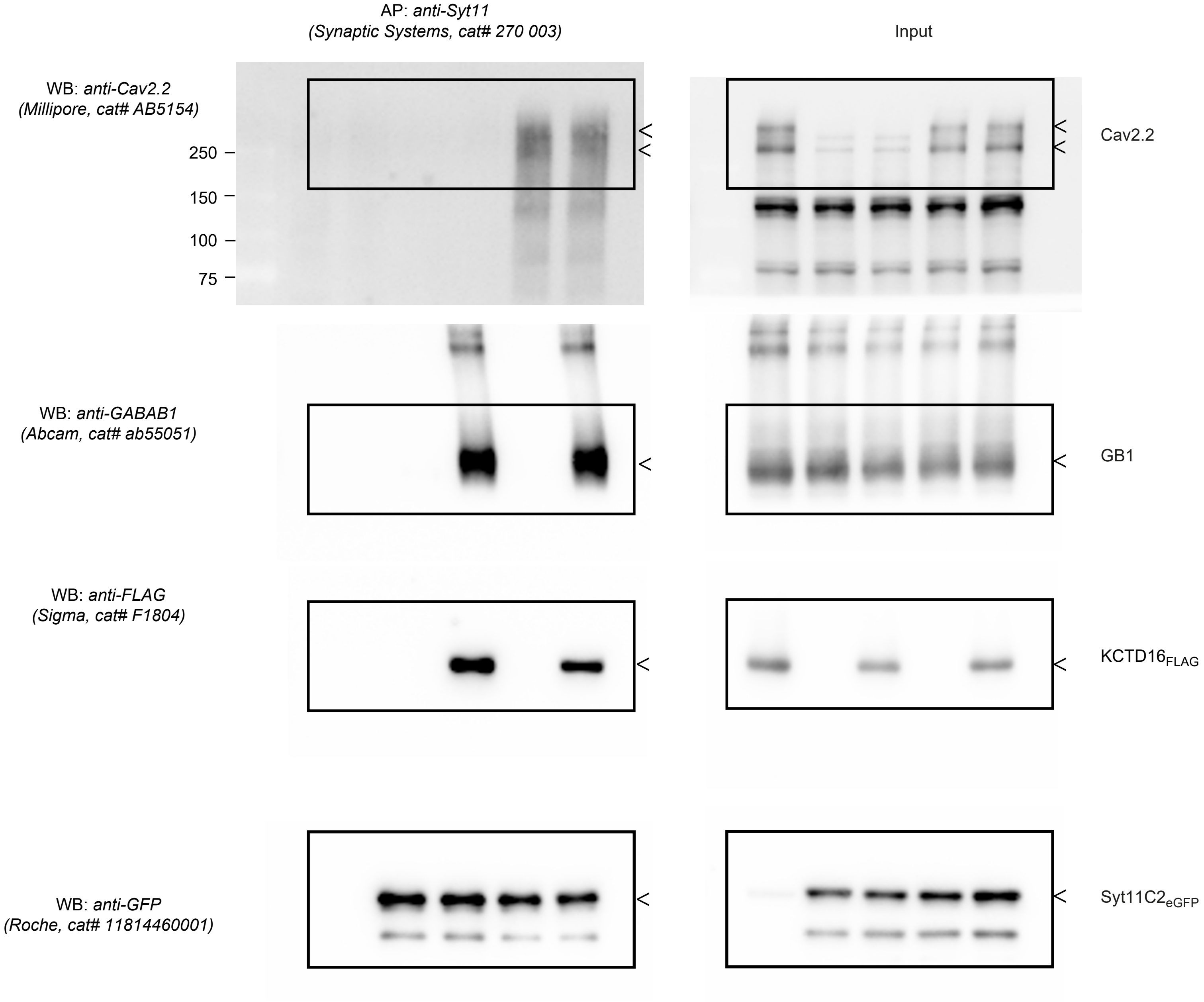
AP: anti-Syt11 (Synaptic Systems, cat# 270 003)
Input
WB: anti-Cav2.2 (Millipore, cat# AB5154)
250
150
100
75
Cav2.2
WB: anti-GABAB1 (Abcam, cat# ab55051)
GB1
WB: anti-FLAG (Sigma, cat# F1804)
$KCTD16_{FLAG}$
WB: anti-GFP (Roche, cat# 11814460001)
$Syt11C2_{eGFP}$
