# Supplementary figures and images for: Synaptotagmin-11 facilitates assembly of a presynaptic signaling complex in post-Golgi cargo vesicles
Source: EMBO Rep. 2024 May 2;25(6):10. doi: 10.1038/s44319-024-00147-0 (PMC11169412; doi:10.1038/s44319-024-00147-0)

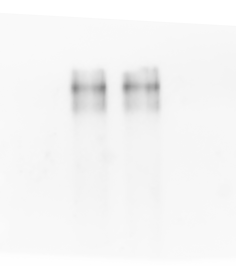

Supplement: Supplementary file 4 — Source data Fig. 1 [file 44319_2024_147_MOESM4_ESM.zip › EMBOR-2023-58002V2_SourceDataForFigure1/1B/1B bottom/AP_western_Cav2.2.tif]

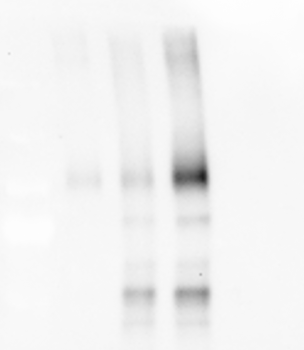

Supplement: Supplementary file 4 — Source data Fig. 1 [file 44319_2024_147_MOESM4_ESM.zip › EMBOR-2023-58002V2_SourceDataForFigure1/1B/1B bottom/AP_western_GB1.tif]

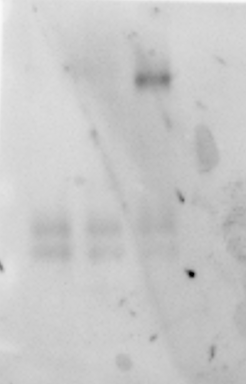

Supplement: Supplementary file 4 — Source data Fig. 1 [file 44319_2024_147_MOESM4_ESM.zip › EMBOR-2023-58002V2_SourceDataForFigure1/1B/1B bottom/AP_western_KCTD16-FLAG.tif]

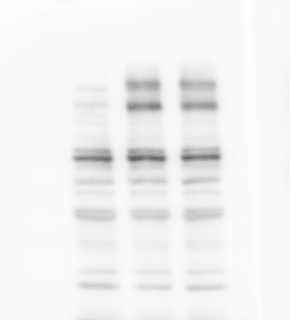

Supplement: Supplementary file 4 — Source data Fig. 1 [file 44319_2024_147_MOESM4_ESM.zip › EMBOR-2023-58002V2_SourceDataForFigure1/1B/1B bottom/Input_western_Cav2.2.tif]

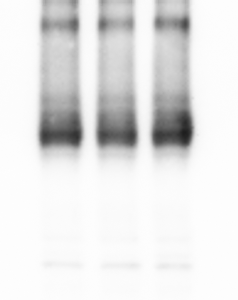

Supplement: Supplementary file 4 — Source data Fig. 1 [file 44319_2024_147_MOESM4_ESM.zip › EMBOR-2023-58002V2_SourceDataForFigure1/1B/1B bottom/Input_western_GB1.tif]

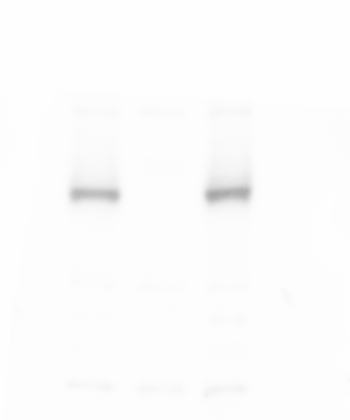

Supplement: Supplementary file 4 — Source data Fig. 1 [file 44319_2024_147_MOESM4_ESM.zip › EMBOR-2023-58002V2_SourceDataForFigure1/1B/1B bottom/Input_western_KCTD16-FLAG.tif]

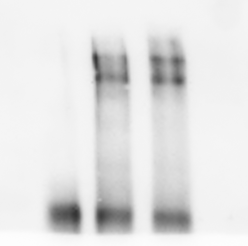

Supplement: Supplementary file 4 — Source data Fig. 1 [file 44319_2024_147_MOESM4_ESM.zip › EMBOR-2023-58002V2_SourceDataForFigure1/1B/1B top/AP_western_Cav2.2.tif]

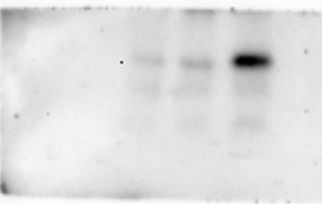

Supplement: Supplementary file 4 — Source data Fig. 1 [file 44319_2024_147_MOESM4_ESM.zip › EMBOR-2023-58002V2_SourceDataForFigure1/1B/1B top/AP_western_GB2ICD-Myc.tif]

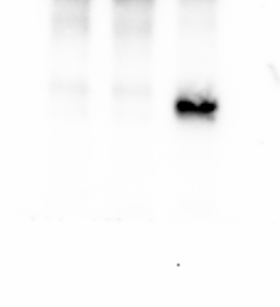

Supplement: Supplementary file 4 — Source data Fig. 1 [file 44319_2024_147_MOESM4_ESM.zip › EMBOR-2023-58002V2_SourceDataForFigure1/1B/1B top/AP_western_KCTD16-FLAG.tif]

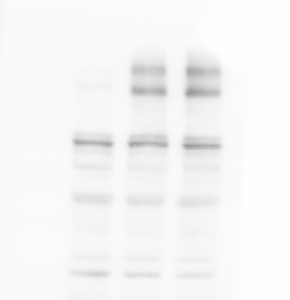

Supplement: Supplementary file 4 — Source data Fig. 1 [file 44319_2024_147_MOESM4_ESM.zip › EMBOR-2023-58002V2_SourceDataForFigure1/1B/1B top/Input_western_Cav2.2.tif]

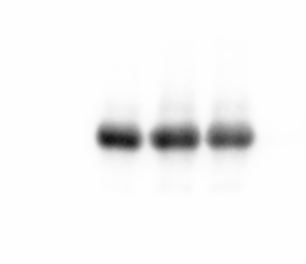

Supplement: Supplementary file 4 — Source data Fig. 1 [file 44319_2024_147_MOESM4_ESM.zip › EMBOR-2023-58002V2_SourceDataForFigure1/1B/1B top/Input_western_GB2ICD-Myc.tif]

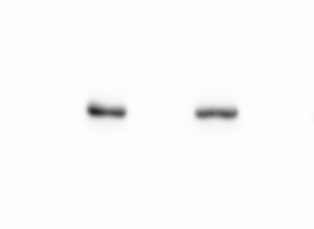

Supplement: Supplementary file 4 — Source data Fig. 1 [file 44319_2024_147_MOESM4_ESM.zip › EMBOR-2023-58002V2_SourceDataForFigure1/1B/1B top/Input_western_KCTD16-FLAG.tif]

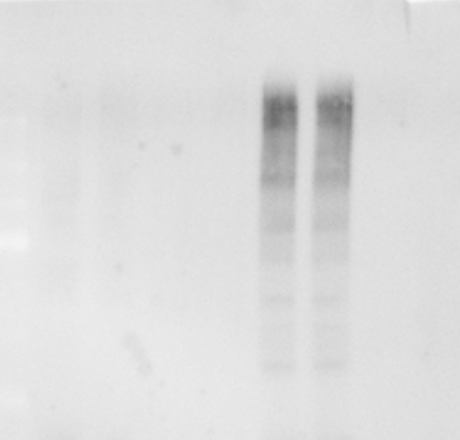

Supplement: Supplementary file 4 — Source data Fig. 1 [file 44319_2024_147_MOESM4_ESM.zip › EMBOR-2023-58002V2_SourceDataForFigure1/1C/AP_western_Cav2.2.tif]

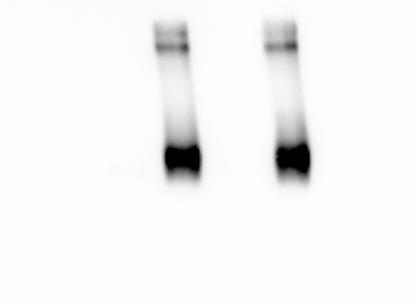

Supplement: Supplementary file 4 — Source data Fig. 1 [file 44319_2024_147_MOESM4_ESM.zip › EMBOR-2023-58002V2_SourceDataForFigure1/1C/AP_western_GB1.tif]

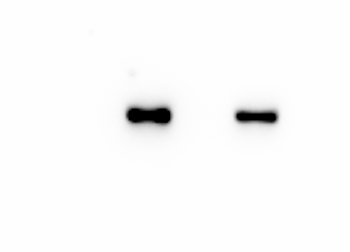

Supplement: Supplementary file 4 — Source data Fig. 1 [file 44319_2024_147_MOESM4_ESM.zip › EMBOR-2023-58002V2_SourceDataForFigure1/1C/AP_western_KCTD16-FLAG.tif]

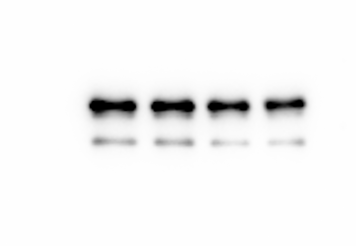

Supplement: Supplementary file 4 — Source data Fig. 1 [file 44319_2024_147_MOESM4_ESM.zip › EMBOR-2023-58002V2_SourceDataForFigure1/1C/AP_western_Syt11C2-eGFP.tif]

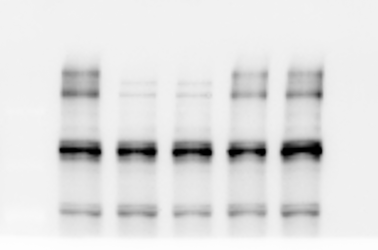

Supplement: Supplementary file 4 — Source data Fig. 1 [file 44319_2024_147_MOESM4_ESM.zip › EMBOR-2023-58002V2_SourceDataForFigure1/1C/Input_western_Cav2.2.tif]

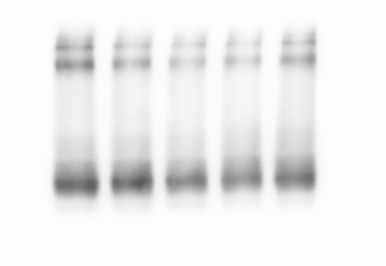

Supplement: Supplementary file 4 — Source data Fig. 1 [file 44319_2024_147_MOESM4_ESM.zip › EMBOR-2023-58002V2_SourceDataForFigure1/1C/Input_western_GB1.tif]

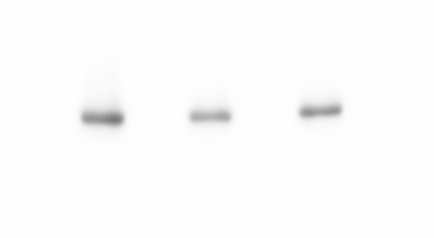

Supplement: Supplementary file 4 — Source data Fig. 1 [file 44319_2024_147_MOESM4_ESM.zip › EMBOR-2023-58002V2_SourceDataForFigure1/1C/Input_western_KCTD16-FLAG.tif]

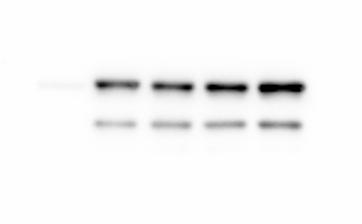

Supplement: Supplementary file 4 — Source data Fig. 1 [file 44319_2024_147_MOESM4_ESM.zip › EMBOR-2023-58002V2_SourceDataForFigure1/1C/Input_western_Syt11C2-eGFP.tif]

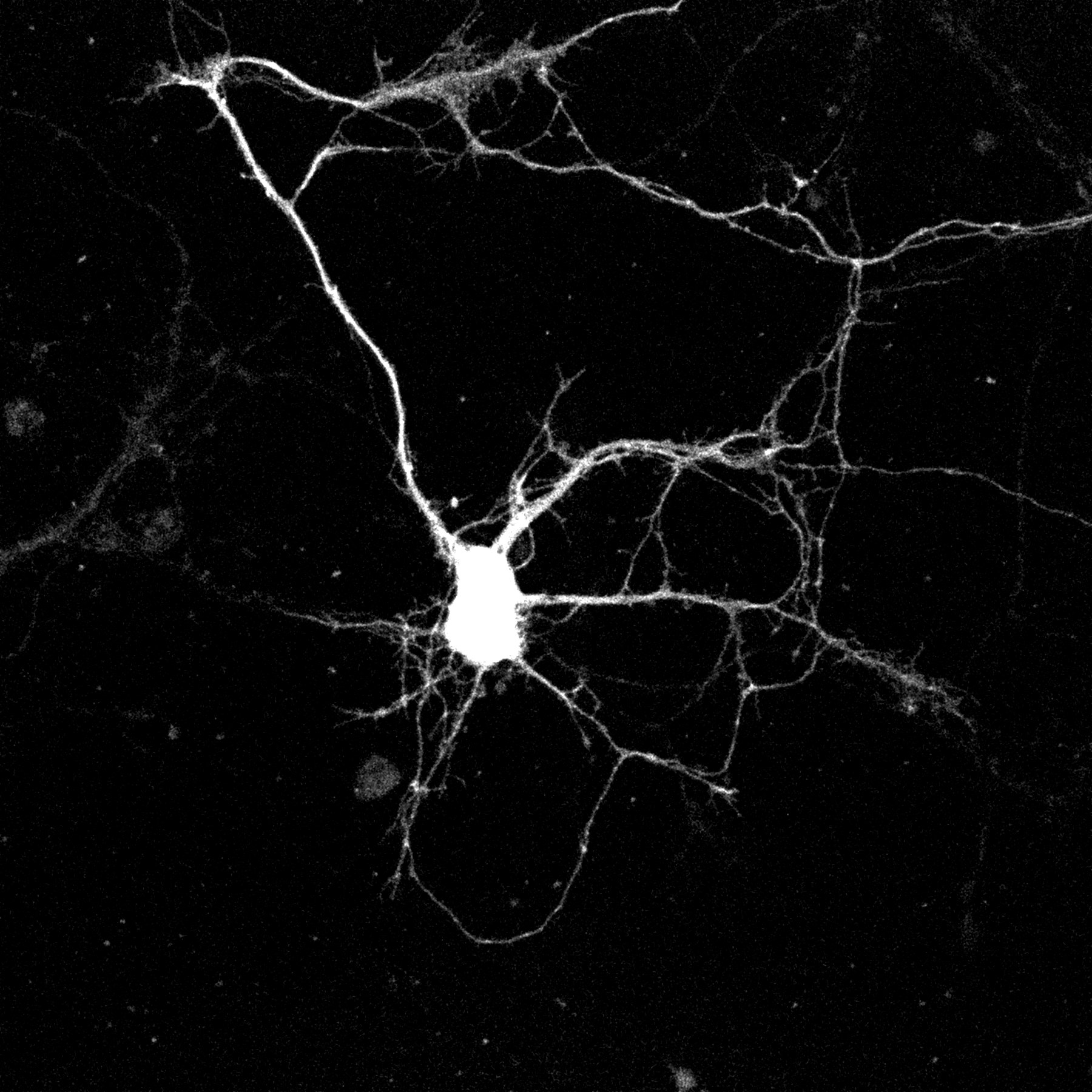

Supplement: Supplementary file 6 — Source data Fig. 3 [file 44319_2024_147_MOESM6_ESM.zip › EMBOR-2023-58002V2_SourceDataForFigure3/3B/Kctd16-KO_mCherry.tif]

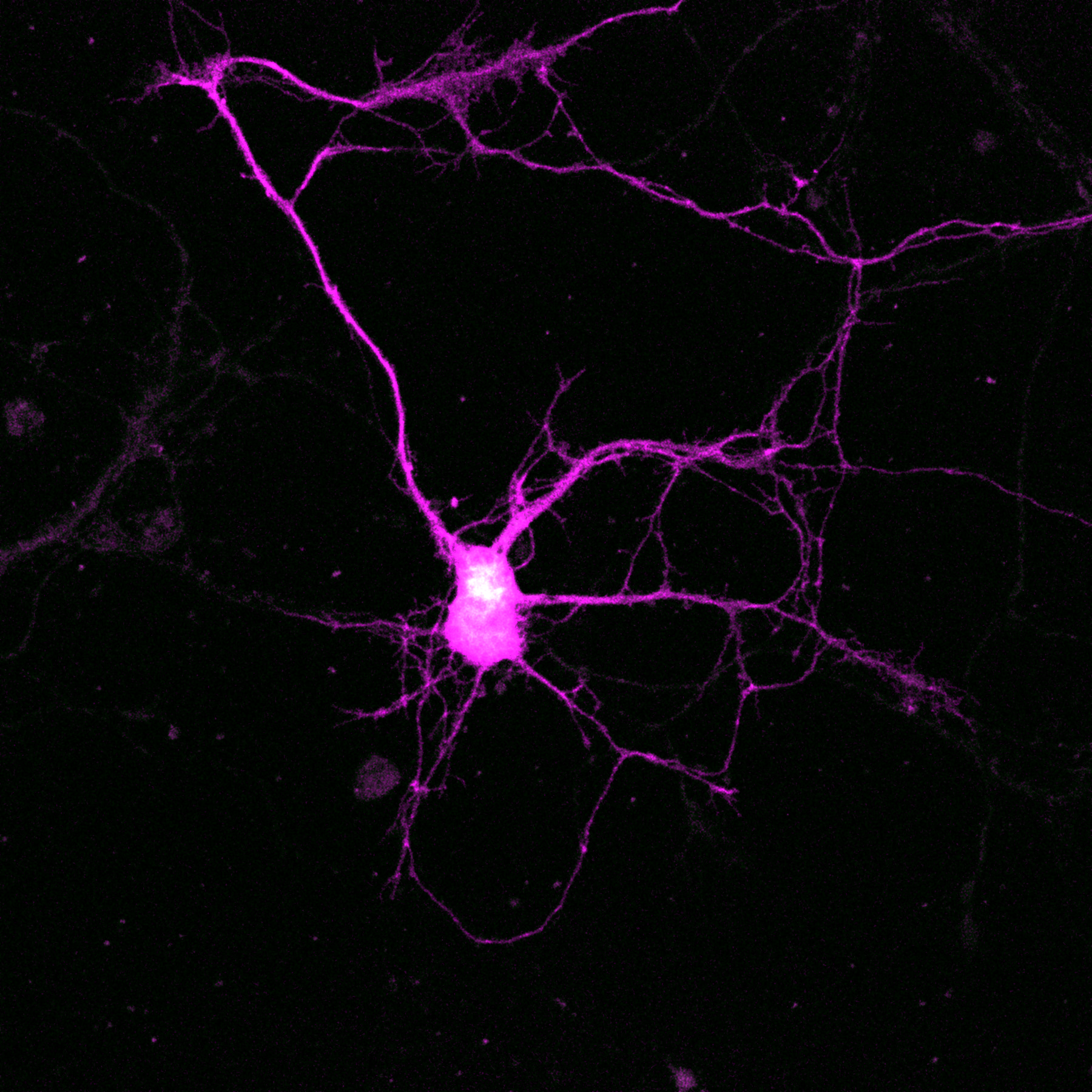

Supplement: Supplementary file 6 — Source data Fig. 3 [file 44319_2024_147_MOESM6_ESM.zip › EMBOR-2023-58002V2_SourceDataForFigure3/3B/Kctd16-KO_Merge.tif]

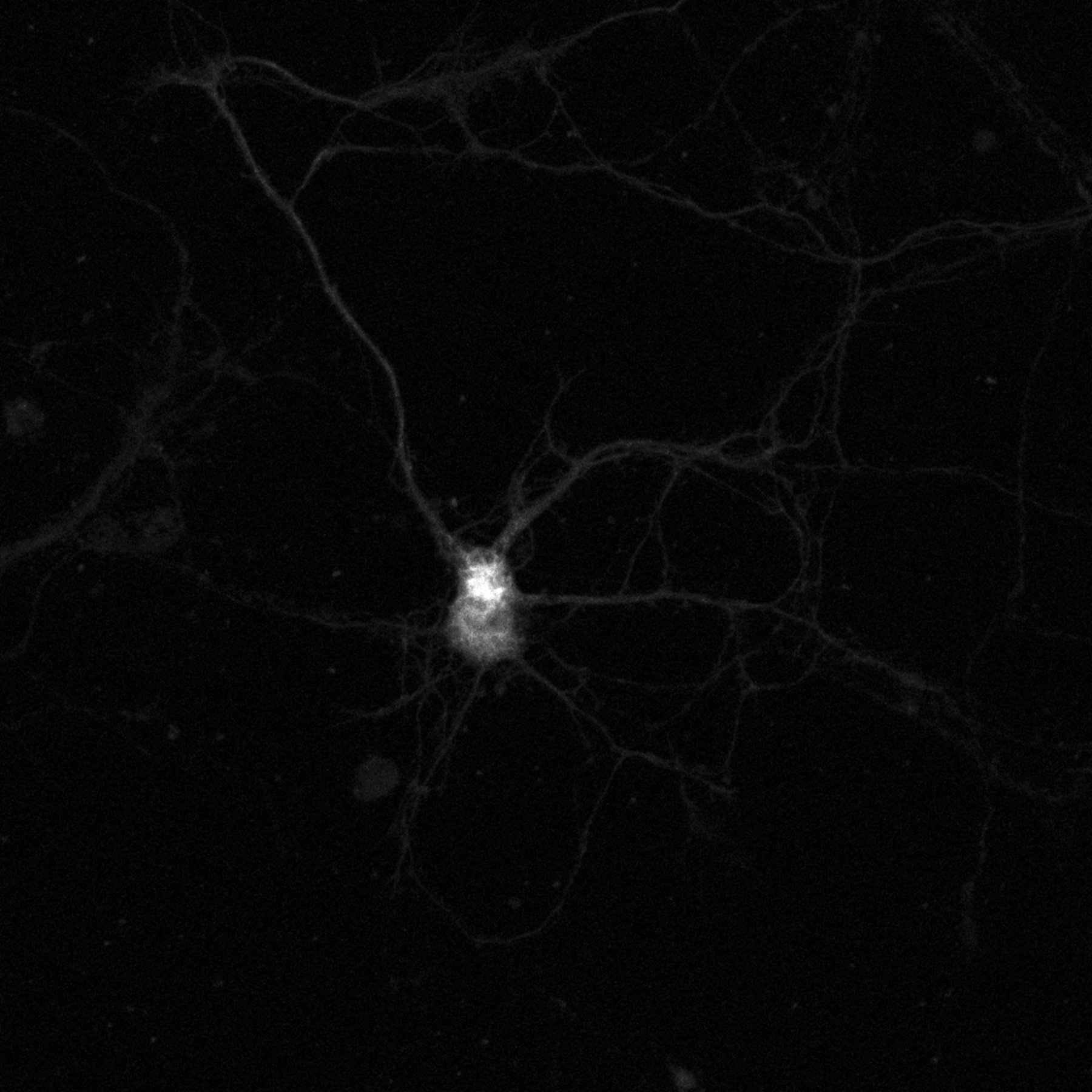

Supplement: Supplementary file 6 — Source data Fig. 3 [file 44319_2024_147_MOESM6_ESM.zip › EMBOR-2023-58002V2_SourceDataForFigure3/3B/Kctd16-KO_Venus.tif]

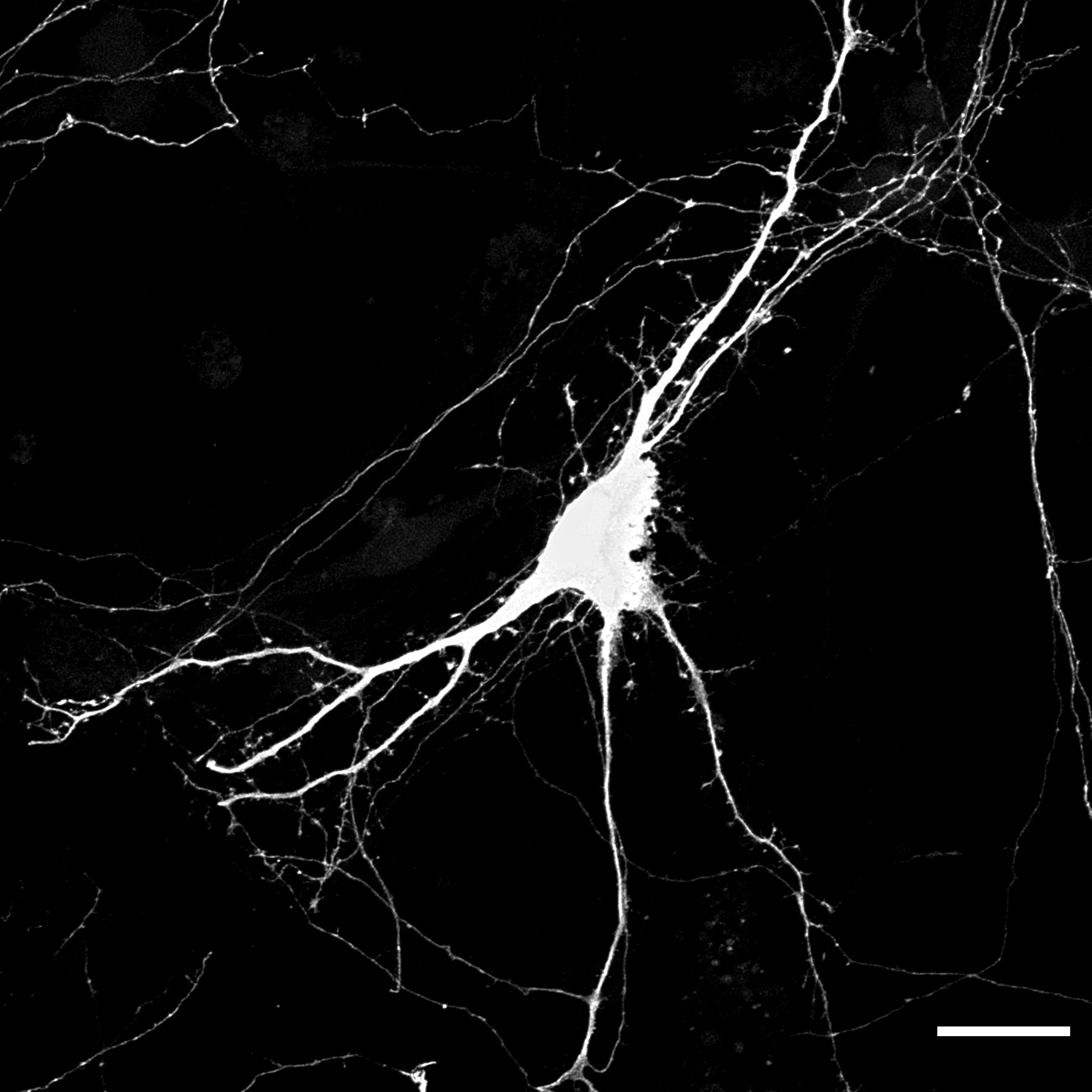

Supplement: Supplementary file 6 — Source data Fig. 3 [file 44319_2024_147_MOESM6_ESM.zip › EMBOR-2023-58002V2_SourceDataForFigure3/3B/Kctd16-WT_mCherry.tif]

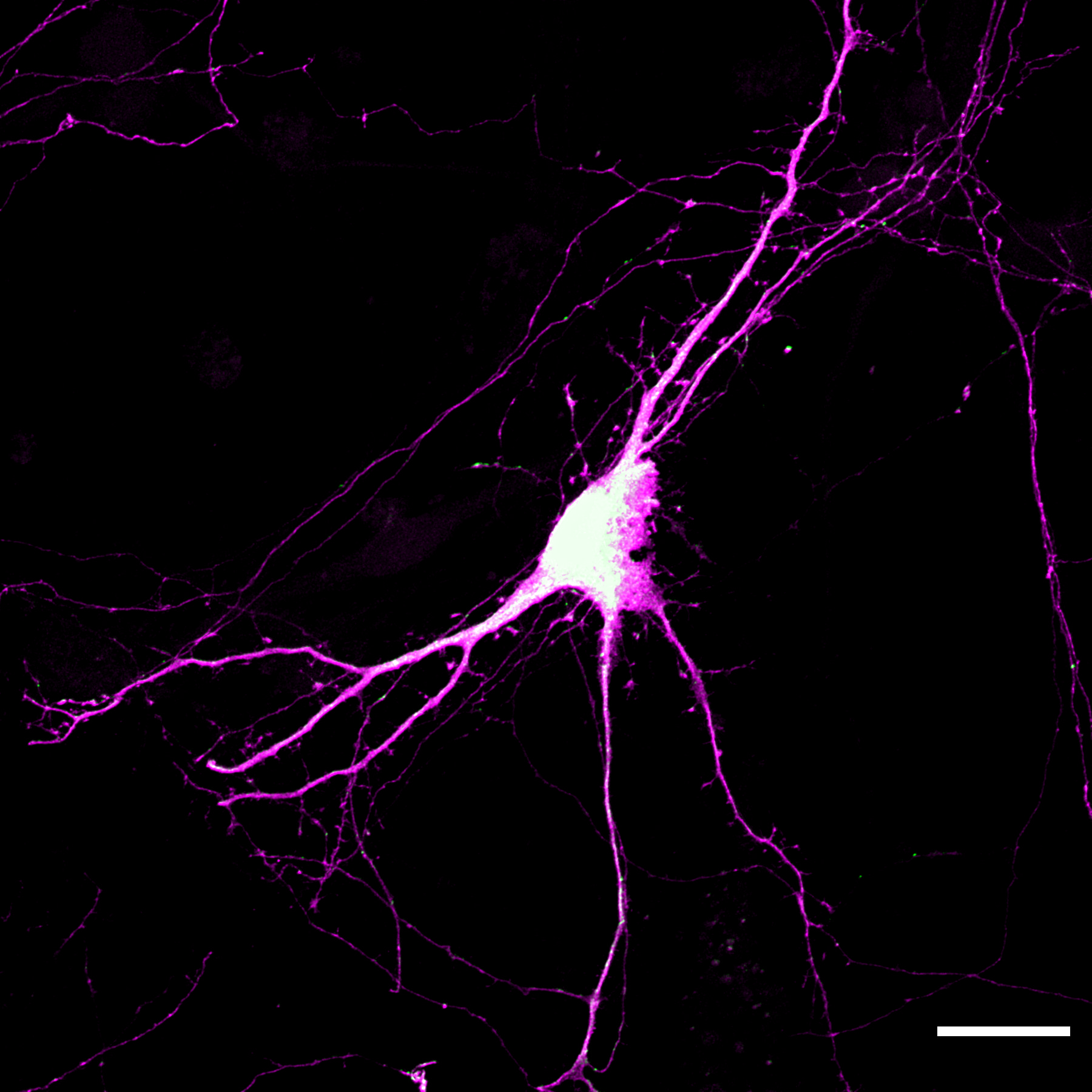

Supplement: Supplementary file 6 — Source data Fig. 3 [file 44319_2024_147_MOESM6_ESM.zip › EMBOR-2023-58002V2_SourceDataForFigure3/3B/Kctd16-WT_Merge.tif]

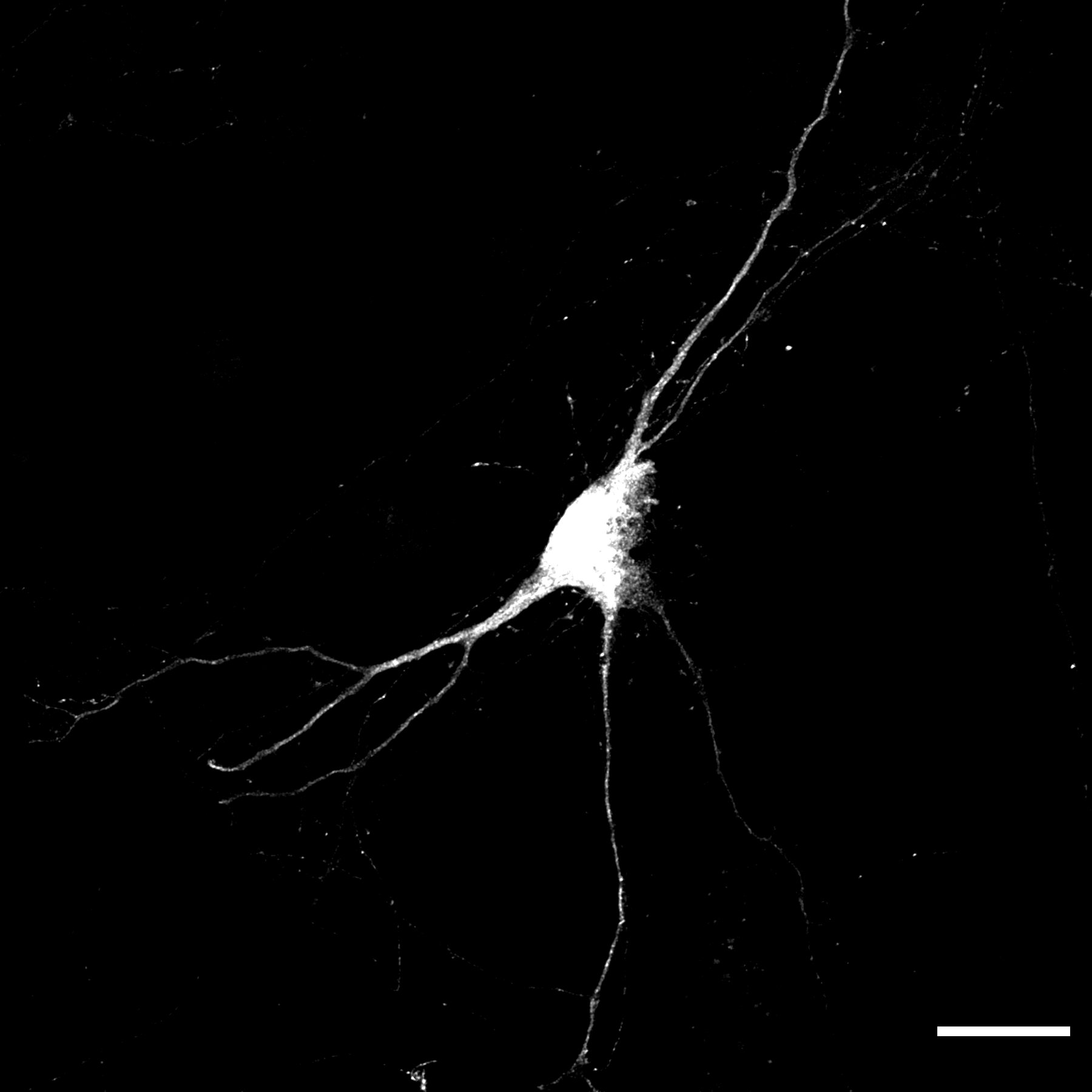

Supplement: Supplementary file 6 — Source data Fig. 3 [file 44319_2024_147_MOESM6_ESM.zip › EMBOR-2023-58002V2_SourceDataForFigure3/3B/Kctd16-WT_Venus.tif]

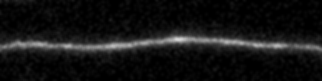

Supplement: Supplementary file 6 — Source data Fig. 3 [file 44319_2024_147_MOESM6_ESM.zip › EMBOR-2023-58002V2_SourceDataForFigure3/3C/Axon_mCherry.tif]

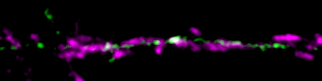

Supplement: Supplementary file 6 — Source data Fig. 3 [file 44319_2024_147_MOESM6_ESM.zip › EMBOR-2023-58002V2_SourceDataForFigure3/3C/Axon_Merge.tif]

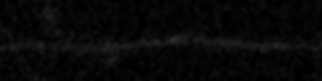

Supplement: Supplementary file 6 — Source data Fig. 3 [file 44319_2024_147_MOESM6_ESM.zip › EMBOR-2023-58002V2_SourceDataForFigure3/3C/Axon_Venus.tif]

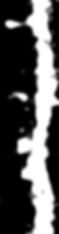

Supplement: Supplementary file 6 — Source data Fig. 3 [file 44319_2024_147_MOESM6_ESM.zip › EMBOR-2023-58002V2_SourceDataForFigure3/3C/Dendrite_mCherry.tif]

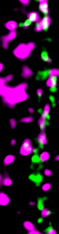

Supplement: Supplementary file 6 — Source data Fig. 3 [file 44319_2024_147_MOESM6_ESM.zip › EMBOR-2023-58002V2_SourceDataForFigure3/3C/Dendrite_Merge.tif]

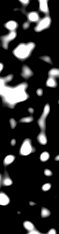

Supplement: Supplementary file 6 — Source data Fig. 3 [file 44319_2024_147_MOESM6_ESM.zip › EMBOR-2023-58002V2_SourceDataForFigure3/3C/Dendrite_PSD95.tif]

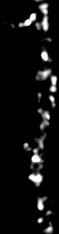

Supplement: Supplementary file 6 — Source data Fig. 3 [file 44319_2024_147_MOESM6_ESM.zip › EMBOR-2023-58002V2_SourceDataForFigure3/3C/Dendrite_Venus.tif]

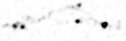

Supplement: Supplementary file 6 — Source data Fig. 3 [file 44319_2024_147_MOESM6_ESM.zip › EMBOR-2023-58002V2_SourceDataForFigure3/3E/0 min.tif]

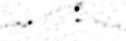

Supplement: Supplementary file 6 — Source data Fig. 3 [file 44319_2024_147_MOESM6_ESM.zip › EMBOR-2023-58002V2_SourceDataForFigure3/3E/1 min.tif]

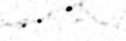

Supplement: Supplementary file 6 — Source data Fig. 3 [file 44319_2024_147_MOESM6_ESM.zip › EMBOR-2023-58002V2_SourceDataForFigure3/3E/2 min.tif]

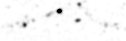

Supplement: Supplementary file 6 — Source data Fig. 3 [file 44319_2024_147_MOESM6_ESM.zip › EMBOR-2023-58002V2_SourceDataForFigure3/3E/3 min.tif]

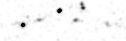

Supplement: Supplementary file 6 — Source data Fig. 3 [file 44319_2024_147_MOESM6_ESM.zip › EMBOR-2023-58002V2_SourceDataForFigure3/3E/4 min.tif]

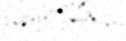

Supplement: Supplementary file 6 — Source data Fig. 3 [file 44319_2024_147_MOESM6_ESM.zip › EMBOR-2023-58002V2_SourceDataForFigure3/3E/5 min.tif]

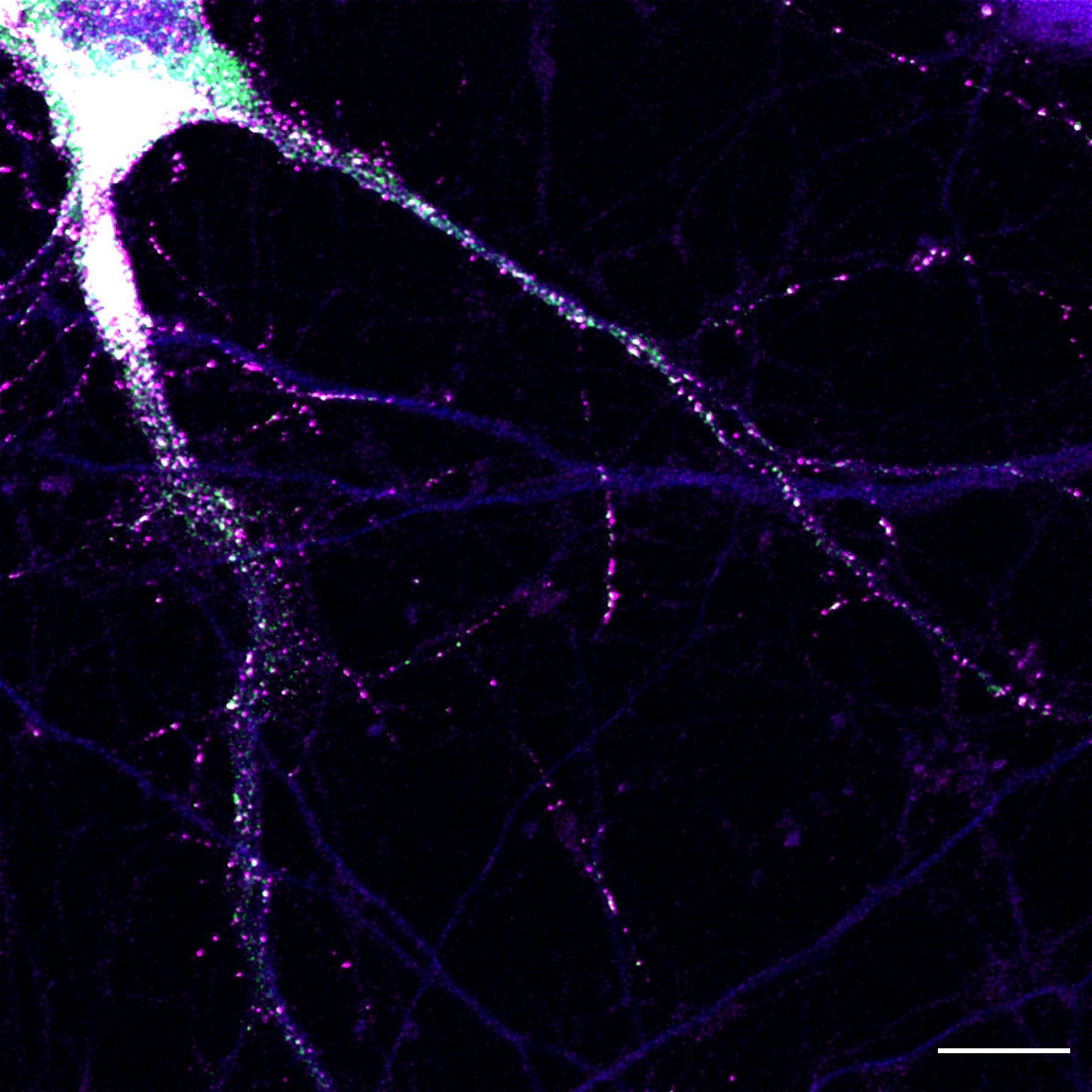

Supplement: Supplementary file 6 — Source data Fig. 3 [file 44319_2024_147_MOESM6_ESM.zip › EMBOR-2023-58002V2_SourceDataForFigure3/3F/NPY-mCherry_MAP2.tif]

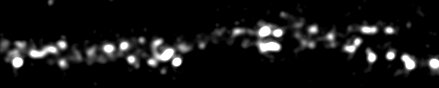

Supplement: Supplementary file 6 — Source data Fig. 3 [file 44319_2024_147_MOESM6_ESM.zip › EMBOR-2023-58002V2_SourceDataForFigure3/3F/NPY_mCherry.tif]

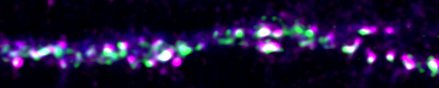

Supplement: Supplementary file 6 — Source data Fig. 3 [file 44319_2024_147_MOESM6_ESM.zip › EMBOR-2023-58002V2_SourceDataForFigure3/3F/NPY_Merge.tif]

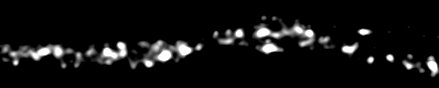

Supplement: Supplementary file 6 — Source data Fig. 3 [file 44319_2024_147_MOESM6_ESM.zip › EMBOR-2023-58002V2_SourceDataForFigure3/3F/NPY_Venus.tif]

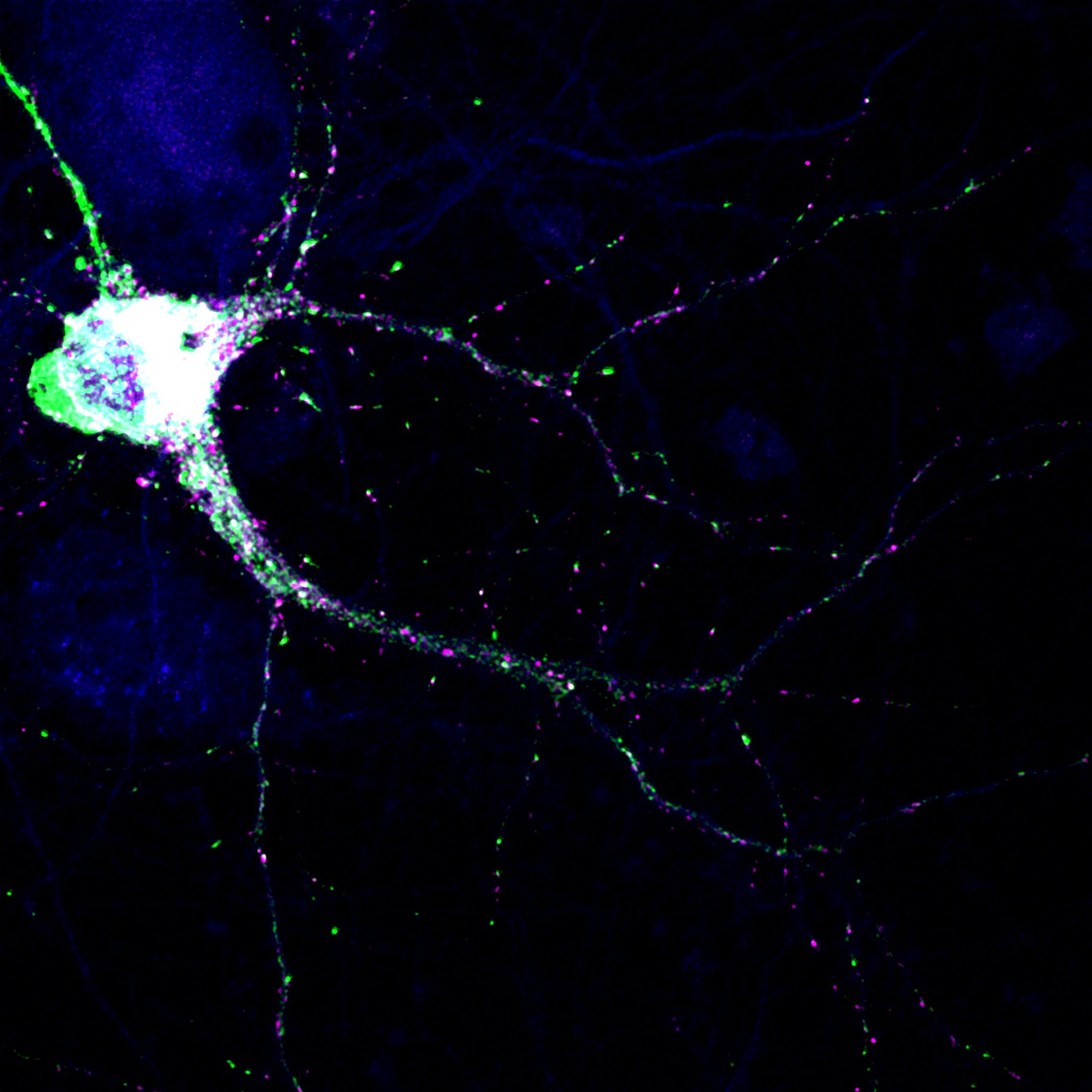

Supplement: Supplementary file 6 — Source data Fig. 3 [file 44319_2024_147_MOESM6_ESM.zip › EMBOR-2023-58002V2_SourceDataForFigure3/3F/Rab5-mCherry_MAP2.tif]

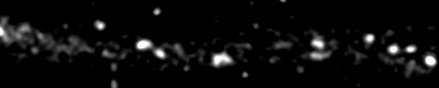

Supplement: Supplementary file 6 — Source data Fig. 3 [file 44319_2024_147_MOESM6_ESM.zip › EMBOR-2023-58002V2_SourceDataForFigure3/3F/Rab5_mCherry.tif]

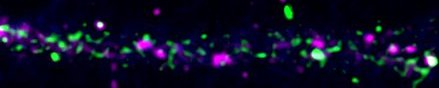

Supplement: Supplementary file 6 — Source data Fig. 3 [file 44319_2024_147_MOESM6_ESM.zip › EMBOR-2023-58002V2_SourceDataForFigure3/3F/Rab5_Merge.tif]

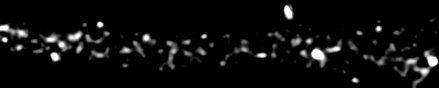

Supplement: Supplementary file 6 — Source data Fig. 3 [file 44319_2024_147_MOESM6_ESM.zip › EMBOR-2023-58002V2_SourceDataForFigure3/3F/Rab5_Venus.tif]

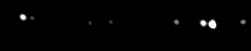

Supplement: Supplementary file 7 — Source data Fig. 4 [file 44319_2024_147_MOESM7_ESM.zip › EMBOR-2023-58002V2_SourceDataForFigure4/4A/Axon_Syt11-KO_Cav2.2.tif]

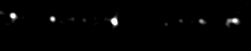

Supplement: Supplementary file 7 — Source data Fig. 4 [file 44319_2024_147_MOESM7_ESM.zip › EMBOR-2023-58002V2_SourceDataForFigure4/4A/Axon_Syt11-KO_GB2.tif]

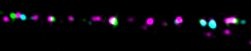

Supplement: Supplementary file 7 — Source data Fig. 4 [file 44319_2024_147_MOESM7_ESM.zip › EMBOR-2023-58002V2_SourceDataForFigure4/4A/Axon_Syt11-KO_Merge.tif]

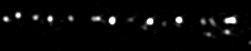

Supplement: Supplementary file 7 — Source data Fig. 4 [file 44319_2024_147_MOESM7_ESM.zip › EMBOR-2023-58002V2_SourceDataForFigure4/4A/Axon_Syt11-KO_NPY.tif]

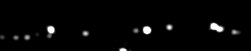

Supplement: Supplementary file 7 — Source data Fig. 4 [file 44319_2024_147_MOESM7_ESM.zip › EMBOR-2023-58002V2_SourceDataForFigure4/4A/Axon_Syt11-WT_Cav2.2.tif]

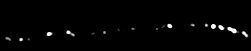

Supplement: Supplementary file 7 — Source data Fig. 4 [file 44319_2024_147_MOESM7_ESM.zip › EMBOR-2023-58002V2_SourceDataForFigure4/4A/Axon_Syt11-WT_GB2.tif]

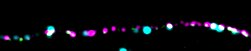

Supplement: Supplementary file 7 — Source data Fig. 4 [file 44319_2024_147_MOESM7_ESM.zip › EMBOR-2023-58002V2_SourceDataForFigure4/4A/Axon_Syt11-WT_Merge.tif]

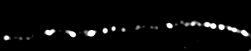

Supplement: Supplementary file 7 — Source data Fig. 4 [file 44319_2024_147_MOESM7_ESM.zip › EMBOR-2023-58002V2_SourceDataForFigure4/4A/Axon_Syt11-WT_NPY.tif]

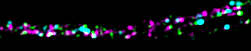

Supplement: Supplementary file 7 — Source data Fig. 4 [file 44319_2024_147_MOESM7_ESM.zip › EMBOR-2023-58002V2_SourceDataForFigure4/4A/Dendrite_Syt11-KO_Merge.tif]

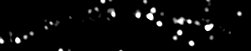

Supplement: Supplementary file 7 — Source data Fig. 4 [file 44319_2024_147_MOESM7_ESM.zip › EMBOR-2023-58002V2_SourceDataForFigure4/4A/Dendrite_Syt11-WT_Cav2.2.tif]

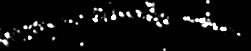

Supplement: Supplementary file 7 — Source data Fig. 4 [file 44319_2024_147_MOESM7_ESM.zip › EMBOR-2023-58002V2_SourceDataForFigure4/4A/Dendrite_Syt11-WT_GB2.tif]

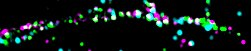

Supplement: Supplementary file 7 — Source data Fig. 4 [file 44319_2024_147_MOESM7_ESM.zip › EMBOR-2023-58002V2_SourceDataForFigure4/4A/Dendrite_Syt11-WT_Merge.tif]

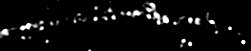

Supplement: Supplementary file 7 — Source data Fig. 4 [file 44319_2024_147_MOESM7_ESM.zip › EMBOR-2023-58002V2_SourceDataForFigure4/4A/Dendrite_Syt11-WT_NPY.tif]

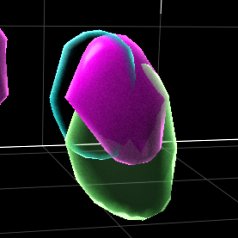

Supplement: Supplementary file 7 — Source data Fig. 4 [file 44319_2024_147_MOESM7_ESM.zip › EMBOR-2023-58002V2_SourceDataForFigure4/4B/GB2+,Cav+.jpg]

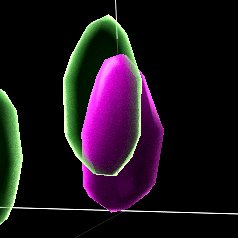

Supplement: Supplementary file 7 — Source data Fig. 4 [file 44319_2024_147_MOESM7_ESM.zip › EMBOR-2023-58002V2_SourceDataForFigure4/4B/GB2+,Cav-.jpg]

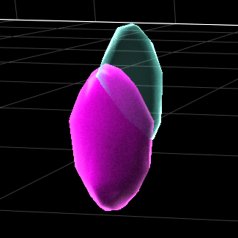

Supplement: Supplementary file 7 — Source data Fig. 4 [file 44319_2024_147_MOESM7_ESM.zip › EMBOR-2023-58002V2_SourceDataForFigure4/4B/GB2-,Cav+.jpg]

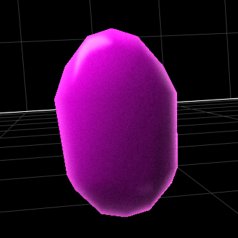

Supplement: Supplementary file 7 — Source data Fig. 4 [file 44319_2024_147_MOESM7_ESM.zip › EMBOR-2023-58002V2_SourceDataForFigure4/4B/GB2-,Cav-.jpg]

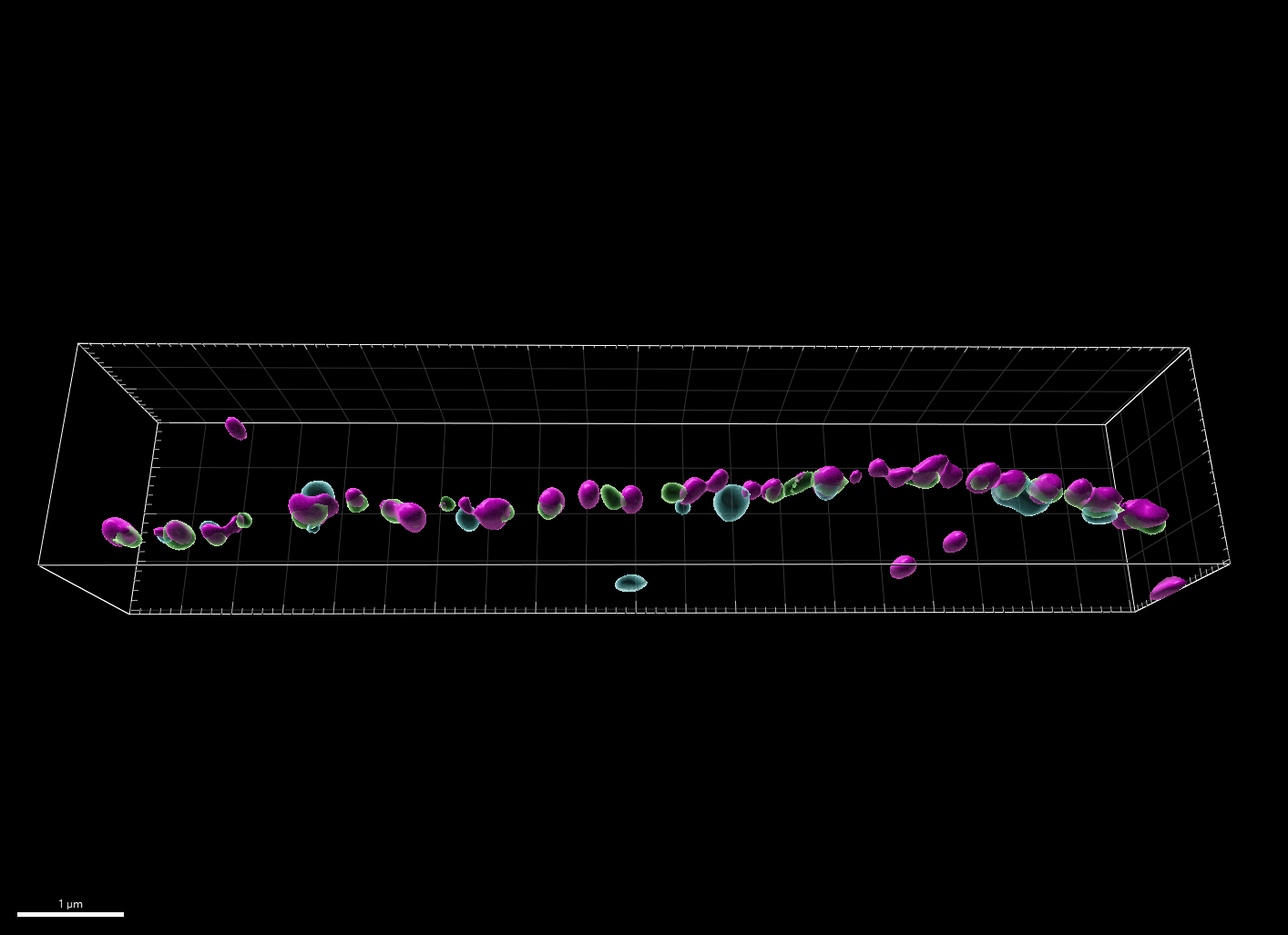

Supplement: Supplementary file 7 — Source data Fig. 4 [file 44319_2024_147_MOESM7_ESM.zip › EMBOR-2023-58002V2_SourceDataForFigure4/4B/NPY,GB2,Cav2.2.png]
