# Supplementary material for: Synaptotagmin-11 facilitates assembly of a presynaptic signaling complex in post-Golgi cargo vesicles
Source: EMBO Rep. 2024 May 2;25(6):10. doi: 10.1038/s44319-024-00147-0 (PMC11169412; doi:10.1038/s44319-024-00147-0)
Supplement: Supplementary file 11 — Expanded View Figures [file 44319_2024_147_MOESM11_ESM.pdf]

Expanded View Figures

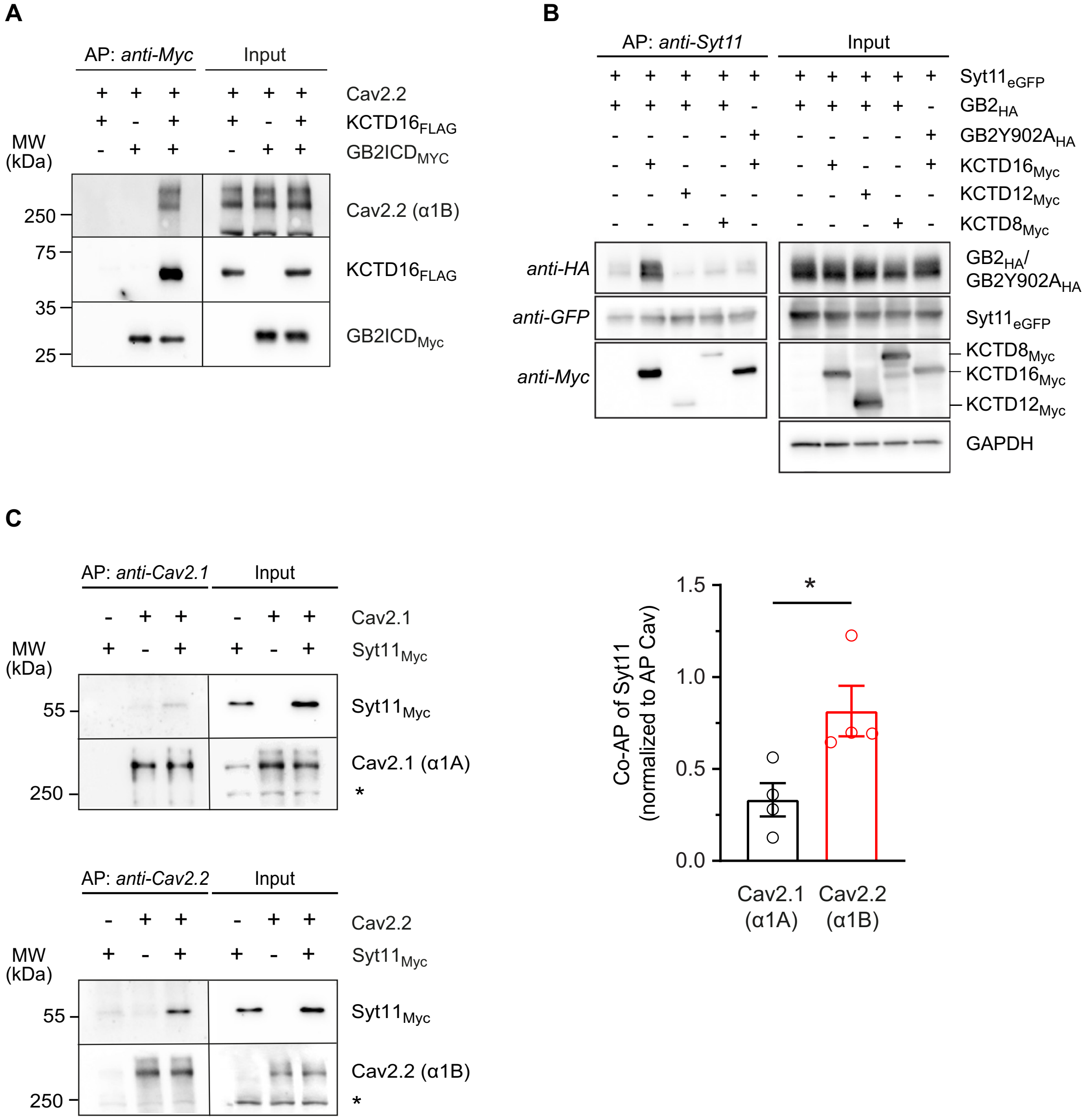

Figure EV1. Mapping of protein-protein interactions within Syt11/GBR/Cav2.2 complexes in HEK293T cells.

(**A**) Cav2.2 co-purifies with the Myc-tagged intracellular C-terminal domain of GB2 (GB2ICD) in the presence of FLAG-tagged KCTD16 from total cell lysates of transfected HEK293T cells. The α1B subunit of Cav2.2 channels was co-expressed with auxiliary β and α2δ subunits. (**B**) HA-tagged GB2 co-purifies with eGFP-tagged Syt11 in the presence of Myc-tagged KCTD16, but not in the presence of KCTD8 or KCTD12 from total cell lysates of transfected HEK293T cells. HA-tagged GB2Y902A, a GB2 mutant that cannot bind KCTD proteins (Schwenk et al, 2010), does not co-purify with eGFP-tagged Syt11 in the presence of KCTD16. (**C**) Significantly increased co-purification of Myc-tagged Syt11 with the α1B subunit of Cav2.2 channels compared to α1A subunit of Cav2.1 channels from total cell lysates of transfected HEK293T cells. Auxiliary β and α2δ subunits were co-expressed with the α1A and α1B subunits. Representative Western blots (left) and quantification from $n = 4$ independent experiments (right). Values are presented as mean ± SEM, $*p = 0.028$, Mann-Whitney U test.

 © The Author(s)

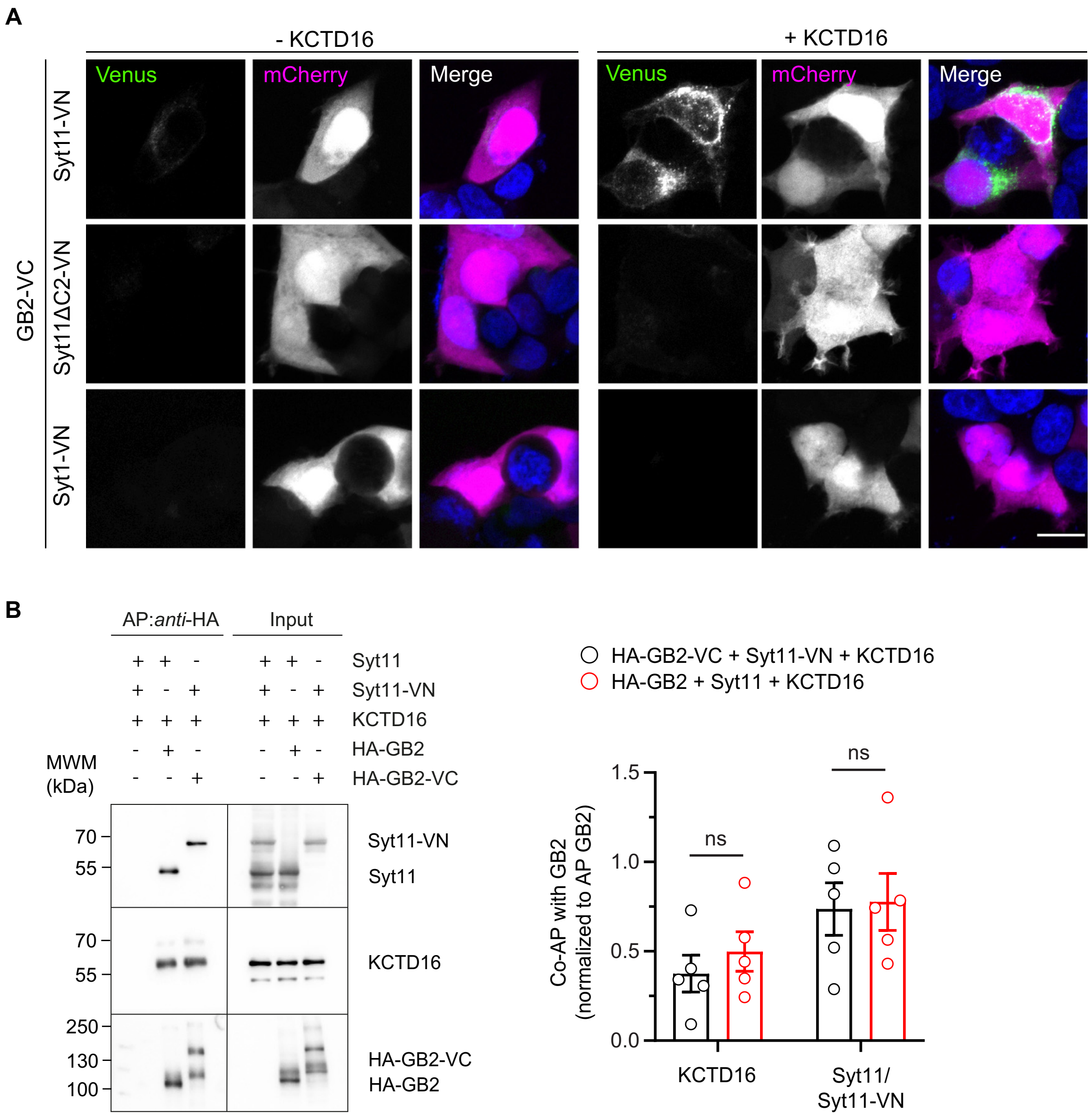

Figure EV2. Validation of the GB2-VC/Syt11-VN BiFC in transfected HEK293T cells.

(**A**) Representative confocal images of HEK293T cells expressing GB2-VC and Syt11-VN tagged with the C-terminal (VC) and N-terminal (VN) fragments of the fluorescent Venus protein (top row). Reconstitution of Venus fluorescence is observed only in cells expressing KCTD16. In control experiments, replacing Syt11-VN with Syt11ΔC2-VN lacking the C2A and C2B domains (middle row) or Syt1-VN (bottom row) does not reconstitute Venus fluorescence. Transfected cells were identified using mCherry. Scale bar: 10 µm. (**B**) Representative Western blots (left) and corresponding quantifications from $n = 5$ independent experiments (right) of APs with anti-HA antibodies from cell lysates of transfected HEK293T cells expressing the indicated constructs. AP and input lanes were probed with anti-Syt11 (top), anti-KCTD16 (middle), and anti-HA (bottom) antibodies. The presence of VN- or VC-tags on Syt11 and GB2, respectively, does not significantly alter the amounts of KCTD16 ($p = 0.436$) and Syt11 ($p = 0.858$) co-purified with GB2. Values are presented as mean ± SEM, ns = not significant, unpaired t-test.

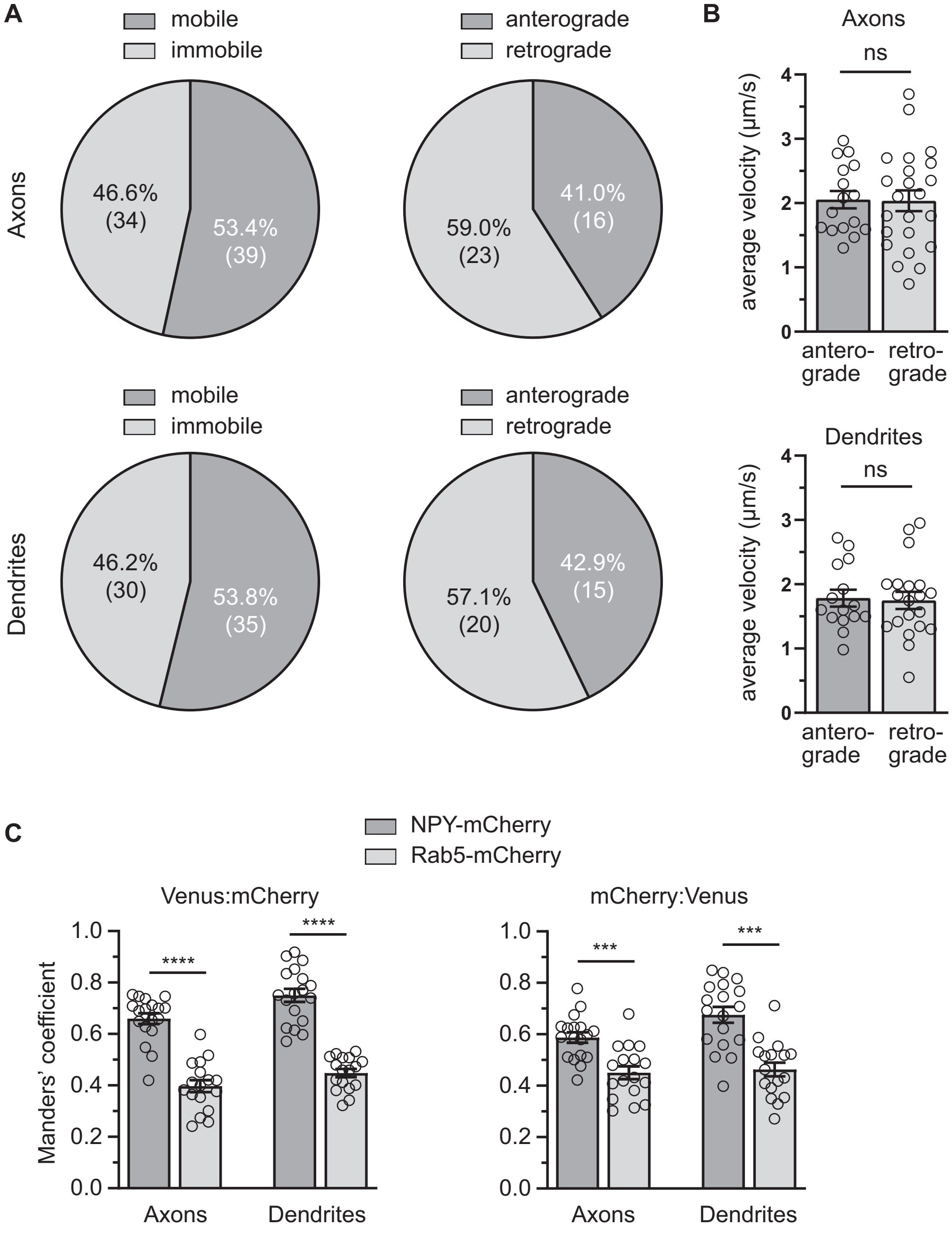

Figure EV3. Trafficking analysis of GB2-VC/Syt11-VN complexes in axons and dendrites of cultured hippocampal neurons.

(**A**) Live-cell imaging analysis of GB2-VC/Syt11-VN complexes (Venus fluorescence) in transfected neurons. Left: Percentage of mobile and immobile complexes. Right: Percentage of complexes traveling antero- and retrograde. The number of complexes analyzed is indicated in brackets. Data are from 4 independent transfections. (**B**) Average velocities of GB2-VC/Syt11-VN complexes traveling antero- and retrograde. Axons: anterograde, $n = 16$ complexes; retrograde, $n = 23$. Dendrites: anterograde, $n = 15$; retrograde, $n = 20$. (**C**) Co-localization of GB2-VC/Syt11-VN complexes and mCherry-tagged NPY or Rab5 in transfected neurons. The Manders' coefficients report the degree of overlap between Venus and mCherry fluorescence. NPY-Cherry, $n = 18$ neurons; Rab5-mCherry, $n = 17$ neurons from 3 independent transfections. Data information: Data are presented as mean ± SEM. Statistical significance was determined by two-way ANOVA (**B**) or Mann–Whitney U test (**C**). ns not significant; ***$p < 0.001$, ****$p < 0.0001$.

© The Author(s)

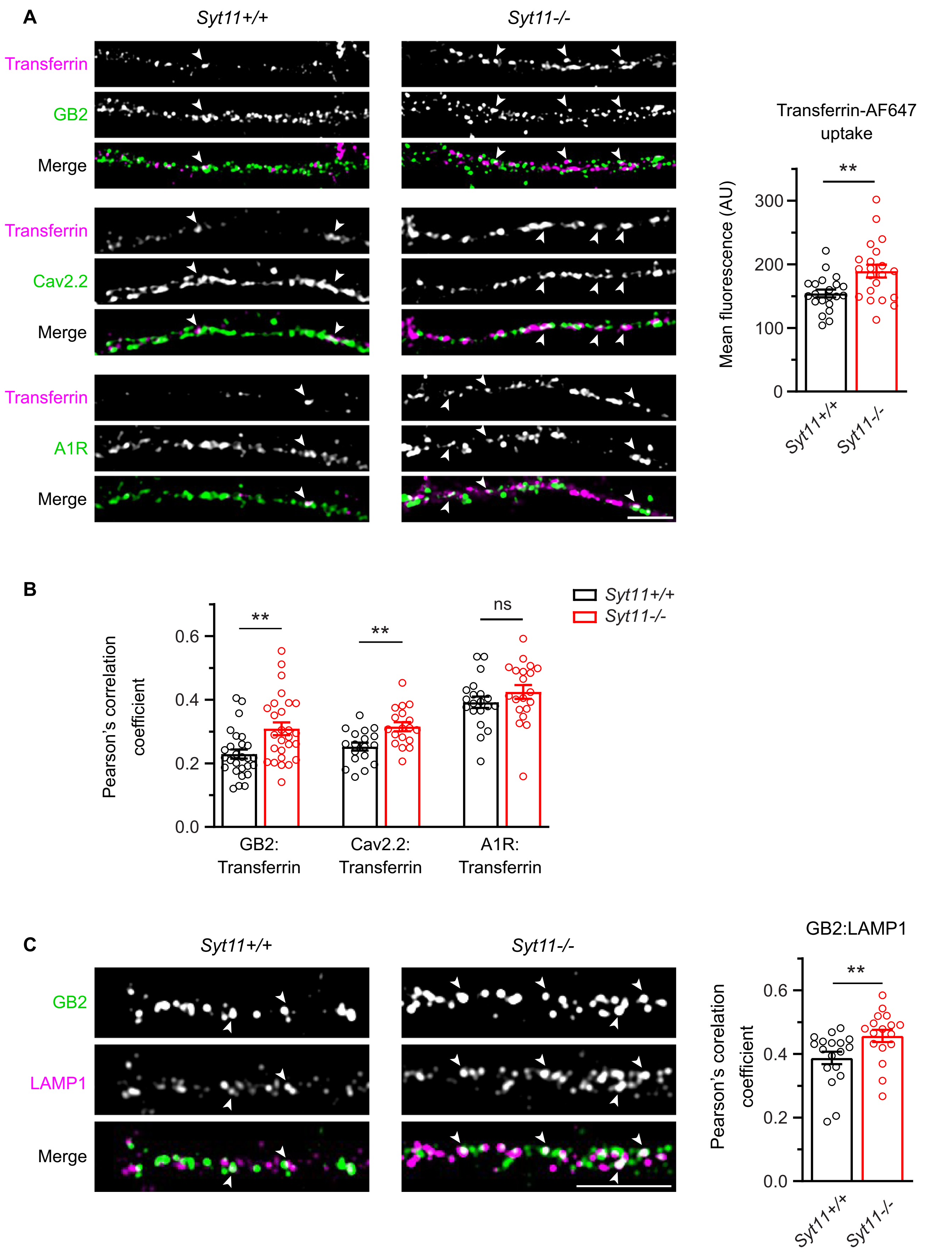
A
Syt11+/+
Syt11-/-
Transferrin
GB2
Merge
Transferrin
Cav2.2
Merge
Transferrin
A1R
Merge
Transferrin-AF647
uptake
Mean fluorescence (AU)
**
300
200
100
0
Syt11+/+
Syt11-/-
B
Pearson's correlation
coefficient
**
**
ns
0.6
0.4
0.2
0.0
GB2:
Transferrin
Cav2.2:
Transferrin
A1R:
Transferrin
Syt11+/+
Syt11-/-
C
Syt11+/+
Syt11-/-
GB2
LAMP1
Merge
GB2:LAMP1
Pearson's corelation
coefficient
**
0.6
0.4
0.2
0.0
Syt11+/+
Syt11-/-

Figure EV4. Syt11 stabilizes GBRs and Cav2.2 channels but not A1R at the cell surface of neurons.

(**A**) Representative confocal images of dendrites of cultured *Syt11*$^{+/+}$ and *Syt11*$^{-/-}$ hippocampal neurons (DIV14). Neurons were incubated with Transferrin-AF647 (magenta) for 30 min to label early endosomes. Fixed and permeabilized neurons were then stained for endogenous GB2, Cav2.2, or A1R (all green). Arrowheads indicate examples of Transferrin-AF647+ vesicles carrying GB2, Cav2.2, or A1R. Scale bar, 5 µm. Increased Transferrin-AF647 uptake is observed in *Syt11*$^{-/-}$ compared to *Syt11*$^{+/+}$ neurons. $n = 21$ neurons for each genotype from 3 independent experiments. (**B**) Quantification of co-localization of GB2, Cav2.2, or A1R with Transferrin-AF647 in experiments described in (**A**). The Pearson's correlation coefficients indicate the degree of co-localization between Transferrin-AF647 and GB2, Cav2.2, or A1R in dendrites. In *Syt11*$^{-/-}$ neurons, co-localization with Transferrin-AF647 is increased for endogenous GB2 and Cav2.2, indicating increased internalization. GB2, $n = 27$ neurons; Cav2.2, $n = 18$ neurons; A1R, $n = 20$ neurons from 3 independent experiments. (**C**) Representative confocal images of dendrites of cultured *Syt11*$^{+/+}$ and *Syt11*$^{-/-}$ hippocampal neurons (DIV14) stained for endogenous GB2 (green) and the lysosome marker LAMP1 (magenta). Arrowheads indicate examples of co-localization of GB2 with LAMP1. Scale bar, 5 µm. Pearson's correlation coefficient indicates increased co-localization of GB2 with LAMP1 in dendrites of *Syt11*$^{-/-}$ neurons. *Syt11*$^{+/+}$, $n = 18$ neurons; *Syt11*$^{-/-}$, $n = 17$ neurons from 3 independent experiments. Data information: Data are presented as mean ± SEM. Statistical significance was determined by Welch's *t*-test (**A**), unpaired Student's *t*-test (**B**), or Mann–Whitney U test (**C**). ns not significant; $^{**}p < 0.01$.

 © The Author(s)

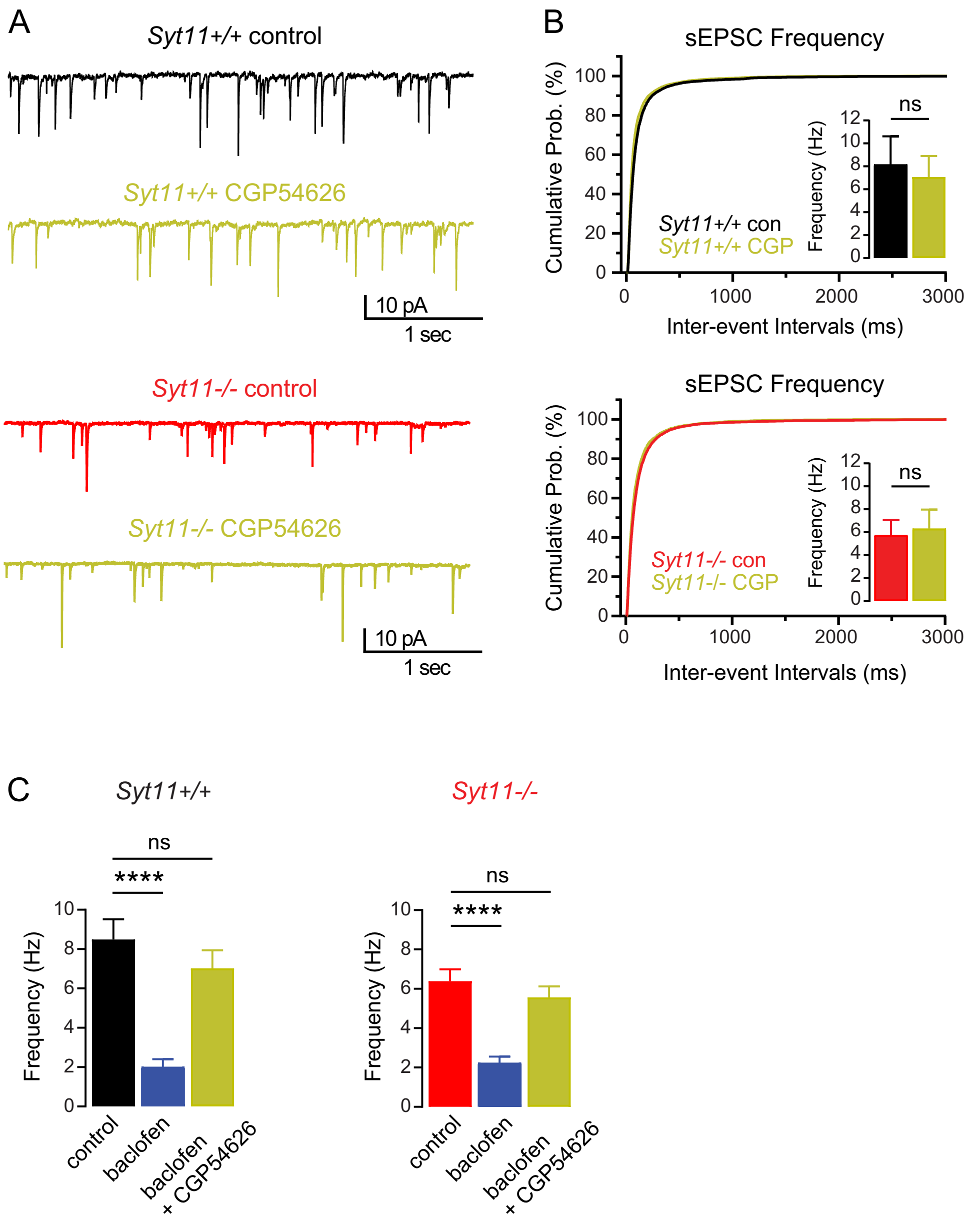

Figure EV5. Lack of tonic or constitutive GBR activity in cultured *Syt11*$^{+/+}$ and *Syt11*$^{-/-}$ hippocampal neurons.

(**A**) Representative traces of sEPSCs recorded from a *Syt11*$^{+/+}$ (top) and *Syt11*$^{-/-}$ (bottom) neuron in the presence of gabazine (10 μM) before (control, black/red) and after application of CGP54626 (4 μM, yellow). (**B**) Cumulative probability distributions of sEPSC inter-event intervals from *Syt11*$^{+/+}$ (top) and *Syt11*$^{-/-}$ (bottom) neurons recorded as in (**A**). In both genotypes, the sEPSC frequency (insets) was not significantly different in the presence of CGP54626 (CGP) compared to control (con). Upper inset: *Syt11*$^{+/+}$ neurons (con: 8.16 ± 2.46 Hz vs CGP: 7.07 ± 1.83 Hz). Lower inset: *Syt11*$^{-/-}$ neurons (con: 5.74 ± 1.32 Hz vs CGP: 6.31 ± 1.67 Hz). $n = 5$ neurons per genotype from 3 preparations. (**C**) Summary bar graph showing the sEPSC frequency of *Syt11*$^{+/+}$ (left) and *Syt11*$^{-/-}$ (right) neurons in the presence of gabazine (10 μM) before (control, black/red) and after application of baclofen (100 μM, blue) and baclofen + CGP54626 (4 μM, yellow). *Syt11*$^{+/+}$, $n = 11$ neurons; *Syt11*$^{-/-}$, $n = 16$ neurons from 6 preparations. Data information: Data are presented as mean ± SEM. Statistical significance was determined by Wilcoxon matched-pairs signed-rank test (**B**) or Friedman test and Dunn's multiple comparisons (**C**). ns not significant; ****$p < 0.0001$.
